# Secured delivery of basic fibroblast growth factor using human serum albumin-based protein nanoparticles for enhanced wound healing and regeneration

**DOI:** 10.1186/s12951-023-02053-4

**Published:** 2023-09-02

**Authors:** Boram Son, Minju Kim, Hyosub Won, Ara Jung, Jihyun Kim, Yonghoe Koo, Na Kyeong Lee, Seung-Ho Baek, Uiyoung Han, Chun Gwon Park, Heungsoo Shin, Bomi Gweon, Jinmyoung Joo, Hee Ho Park

**Affiliations:** 1 Department of Bioengineering, Hanyang University, Seoul, Republic of Korea; 2 Department of Biomedical Engineering, Ulsan National Institute of Science and Technology (UNIST), Ulsan, Republic of Korea; 3 Department of Mechanical Engineering, Sejong University, Seoul, Republic of Korea; 4 Department of Biomedicine & Health Science, College of Medicine, The Catholic University of Korea, Seoul, Korea; 5 Department of Intelligent Precision Healthcare Convergence, Sungkyunkwan University (SKKU), Suwon, Republic of Korea; 6 Center for Bio-based Chemistry, Korea Research Institute of Chemical Technology (KRICT), Ulsan, Korea; 7 Department of Ophthalmology, Stanford University School of Medicine, Stanford, CA USA; 8 Department of Biomedical Engineering, SKKU Institute for Convergence, Sungkyunkwan University (SKKU), Suwon, Republic of Korea; 9 Materials Research Science and Engineering Center, University of California, San Diego, La Jolla, United States; 10 Center for Genomic Integrity, Institute for Basic Science (IBS), Ulsan, Republic of Korea; 11 Research Institute for Convergence of Basic Science, Hanyang University, Seoul, Republic of Korea

**Keywords:** Basic fibroblast growth factor (bFGF), Human serum albumin (HSA), Protein nanoparticle, Wound healing, Tissue regeneration

## Abstract

**Background:**

Basic fibroblast growth factor (bFGF) is one of the critical components accelerating angiogenesis and tissue regeneration by promoting the migration of dermal fibroblasts and endothelial cells associated with matrix formation and remodeling in wound healing process. However, clinical applications of bFGF are substantially limited by its unstable nature due to rapid decomposition under physiological microenvironment.

**Results:**

In this study, we present the bFGF-loaded human serum albumin nanoparticles (HSA-bFGF NPs) as a means of enhanced stability and sustained release platform during tissue regeneration. Spherical shape of the HSA-bFGF NPs with uniform size distribution (polydispersity index < 0.2) is obtained *via* a simple desolvation and crosslinking process. The HSA-bFGF NPs securely load and release the intact soluble bFGF proteins, thereby significantly enhancing the proliferation and migration activity of human dermal fibroblasts. Myofibroblast-related genes and proteins were also significantly down-regulated, indicating decrease in risk of scar formation. Furthermore, wound healing is accelerated while achieving a highly organized extracellular matrix and enhanced angiogenesis in vivo.

**Conclusion:**

Consequently, the HSA-bFGF NPs are suggested not only as a delivery vehicle but also as a protein stabilizer for effective wound healing and tissue regeneration.

**Supplementary Information:**

The online version contains supplementary material available at 10.1186/s12951-023-02053-4.

## Background

Skin is the largest organ that serves as a barrier to the external environments to protect internal tissues and organs [1–3]. When the skin is spoiled and damaged by physical stimuli or diseases, the wound healing process including hemostasis, inflammation, proliferation, and maturation is sequentially proceeded to regenerate the skin tissues properly [4–8]. Since the delayed wound healing may cause further infection or scarring, rapid skin regeneration, particularly dermal fibroblast proliferation and migration, is essential [9–12]. Among the various cytokines and growth factors that promote tissue regeneration, the basic fibroblasts growth factor (bFGF) is representative because it promotes both proliferation and migration of dermal cells for wound healing [13–16]. However, complicated formation process and limited soluble expression of bFGF in the bacterial synthesis (e.g., *E. coli*) have hampered the large-scale production, followed by restricted uses exclusively in research with high cost [17–19]. In addition, the bFGF has a short half-life (typically less than 8 h), requiring continuous supplement for beneficial tissue regeneration [20, 21]. Therefore, innovative strategies have been required for successful delivery of the bFGF to target tissues.

To address the limitations associated with bFGF, researchers have explored diverse methods and materials for its successful delivery to target tissues. These approaches include hydrogels [22–24], microspheres [25, 26], scaffolds [27, 28], and liposomes [29, 30]. Each method has shown promise in protecting bFGF from degradation and enabling controlled release, but they also come with their respective drawbacks that hinder their widespread clinical adoption. Common limitations observed with the existing delivery methods include burst release, limited control over release kinetics, potential toxicity or immunogenicity of carrier materials, challenges in achieving uniform distribution within target tissues, and difficulty in scaling up production for clinical applications.

In this regard, therapeutic delivery systems using nanoparticles as a carrier have attracted tremendous attention as a translational medical platform for efficient and safe administration of biomolecules and drugs [31–34]. Various therapeutic cargos such as molecular drugs, nucleic acids, and proteins can be loaded onto nanoparticles *via* electrostatic interactions [35, 36], self-assembly [37, 38], or covalent conjugations [39, 40]. Among the diverse components to form the nanoparticles for drug delivery systems, albumin is one of the promising candidates as it has been approved by U.S. Food and Drug Administration (FDA) for clinical uses [41]. Considering the non-toxic and non-immunogenic characteristics, albumin has been widely studied as a drug carrier with extended blood circulation time and a large capacity for various therapeutic payloads through electrostatic interactions [42]. Furthermore, albumin nanoparticles are readily modified by surface ligands for specific cell targeting and long-term stability [43–46]. Since albumin is commercially available in large quantities and can be simply prepared in a moderate condition, the biocompatible albumin-based nanoparticles are suitable for mass production and medical translation [47, 48].

**Fig. 1:**
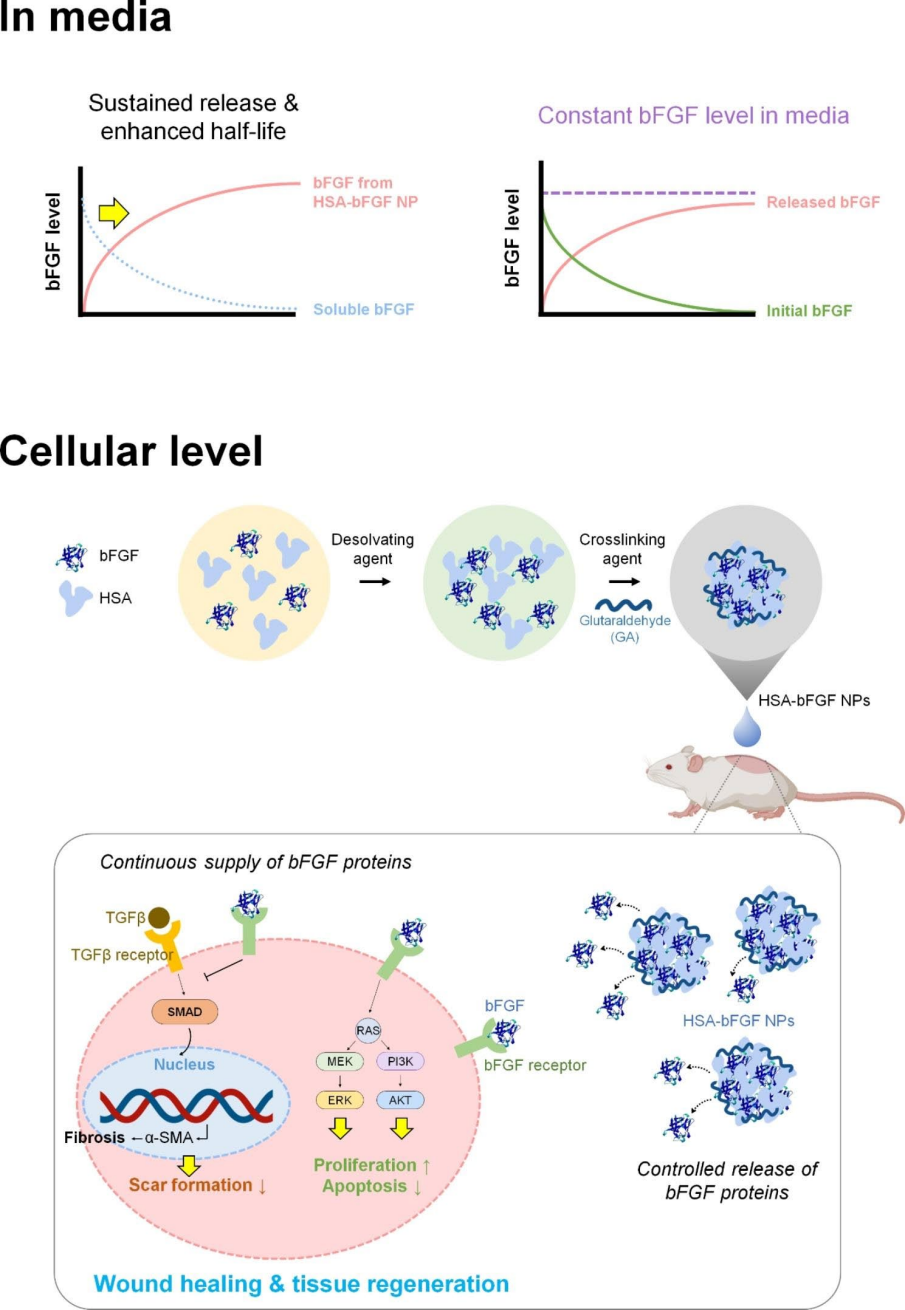
Schematic illustrations depicting cellular delivery of bFGF, followed by wound healing cascades of HSA-bFGF NPs. Intact bFGF has short half-life both in culture media and tissue microenvironment, thus secured crosslinking of bFGF with HSA to form a nanoparticulate is essentially advantageous for long-term supplement of biologically active bFGF in tissue regeneration

Herein, we present the human serum albumin (HSA)-based protein nanoparticles containing bFGF payloads (HSA-bFGF NPs) as a promising carrier for bFGF for improved bFGF stability and continuous supplement for tissue regeneration (Fig. 1). First of all, the aqueously soluble bFGF was obtained from *E. coli* for a mass production by using a vector containing *bFGF-G3* gene mutation, which led the production of soluble bFGF while maintaining the intact activity [49]. The HSA nanoparticles (HSA NPs) were then assembled with corresponding soluble bFGF to form stable nanoparticles, and thoroughly characterized for structural and biological verification. Cell proliferation, migration, and tissue regeneration capability were further systematically investigated by treating to human dermal fibroblasts (HDFs) for in vitro analysis and to injured rat for in vivo wound healing process.

## Results

### Soluble expression and mass production of bFGF protein

**Fig. 2:**
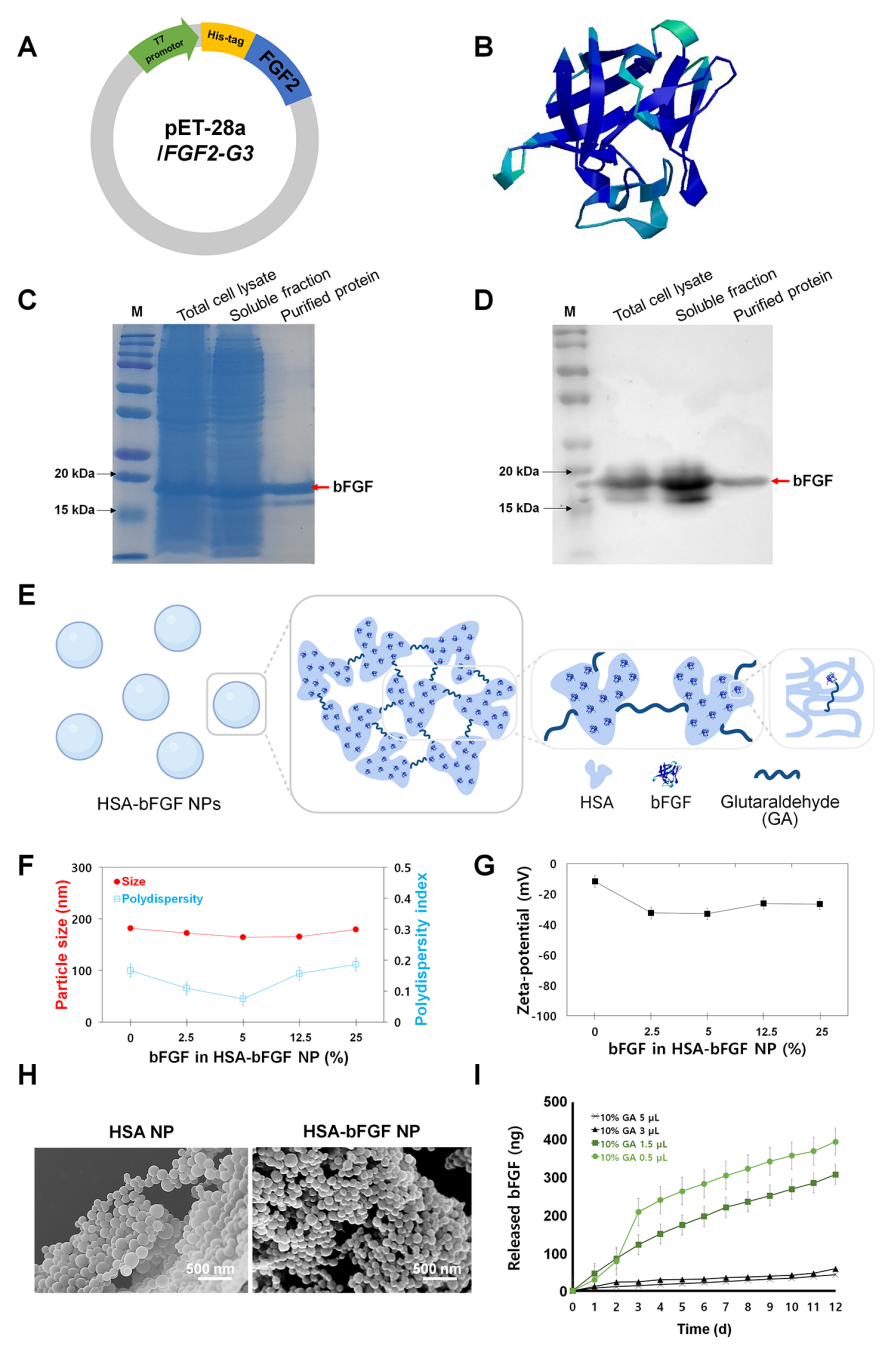
Production of bFGF protein and characterization of the prepared HSA-bFGF NPs. (**A**) Construction of *E. coli* expression vector, pET-28a/*FGF2-G3*. (**B**) Three-dimensional representation structure of bFGF protein. (**C**) SDS-PAGE analysis of the purified recombinant bFGF protein. M means marker. Red arrow indicates bFGF protein. (**D**) Western blot analysis of bFGF protein produced from *E. coli*. M means marker. Red arrow indicates bFGF protein. (**E**) Schematic illustrations demonstrating formation of HSA-bFGF NPs *via* crosslinking between HSA and bFGF mediated by GA. (**F**) Nanoparticle size and PI depending on the bFGF contents (wt%) in HSA-bFGF NPs. (**G**) Zeta-potential changes of the HSA-bFGF NPs. (**H**) SEM images of HSA-bFGF NPs containing 0 or 25 wt% of bFGF protein, respectively. Scale bars, 500 nm. (**I**) In vitro release profile of bFGF from HSA-bFGF NPs depending upon the amount of added crosslinker, GA

For mass production of the recombinant bFGF protein in *E. coli*, the pET-28a/*FGF2-G3* vector mutated for soluble expression of bFGF was used (Fig. 2A). As described in Fig. 2B, three-dimensionally structured bFGF protein was successfully produced in *E. coli*, and then purified with the fast protein liquid chromatography (FPLC). Then the bFGF protein was isolated by 12% sodium dodecyl sulfate-polyacrylamide gel electrophoresis (SDS-PAGE), confirming aqueously soluble expression and size of the bFGF by Coomassie brilliant blue staining (Fig. 2C) and Western blot (Fig. 2D), respectively. According to Fig. 2C, purified bFGF showed the comparable band intensity with those of total cell lysate and soluble fraction, indicating that majority of the bFGF found in total cell lysate was the soluble formulation and successfully purified with the FPLC. As indicated in Western blot (Fig. 2D), the bands of anti-bFGF antibody were observed at 18 kDa, confirming the size of bFGF. To optimize large-scale production process of the soluble bFGF in bacterial synthesis, we conducted two isopropyl-β-D-thiogalactopyranoside (IPTG) induction condition: (1) Fast induction (37 ℃ for 4 h) and (2) slow induction (30 ℃ for 16 h). The final concentration of purified bFGF was 0.1 mg/ml for fast induction while slow induction yields 2.71 mg/ml. Considering the volume of obtained bFGF solution, the total weights of bFGF were 0.8 mg and 32.5 mg in fast induction and slow induction, respectively. Therefore, we confirm that the slow induction process showed substantially higher yields (> 40-fold) of bFGF production than the fast induction.

### Preparation and characterization of HSA-bFGF NPs

The HSA-bFGF NPs were prepared by a desolvation method, in which mixture of HSA and bFGF was gradually dehydrated by adding ethanol drops. Both HSA and bFGF protein molecules became to lose the stability with less hydrated conformation, followed by aggregation with adjacent proteins, resulting in generation of protein nanoparticles. The protein aggregates were then crosslinked with glutaraldehyde (GA) to produce stable formulation (Fig. 2E). Through electrophoretic light scattering (ELS) spectrophotometer, the size, polydispersity index (PI), and zeta-potential of the HSA-bFGF NPs were determined (Fig. 2F and G). The size of HSA-bFGF NPs were consistently obtained in a range of 164 to 182 nm, regardless of the bFGF content ratio (0 to 25%). In addition, the PI of protein nanoparticles was less than 0.2, indicating the narrow size distribution. Zeta-potential of the HSA NPs (no bFGF content) was − 11.8 mV, which decreased to -33 mV with bFGF addition, indicating anionic characteristics of HSA-bFGF NPs and stable dispersion in aqueous solution including deionized water (DIW), phosphate-buffered saline (PBS), and cell culture media. Scanning electron microscope (SEM) images revealed the effect of the bFGF contents on the morphology of protein nanoparticles (Fig. 2H). Compared to the HSA NPs without bFGF, the HSA-bFGF NPs showed slightly diminished morphology while maintaining a comparable size. However, both protein nanoparticles showed a spherical shape with a narrow size distribution regardless of the bFGF content ratio. Overall, the desolvation method leads to a homogeneous HSA NP formation with bFGF proteins as payloads.

Also, in order to investigate degradation of the protein nanoparticles, we measured the size of HSA-bFGF NPs for 14 days (Fig. S1A). Although the diameter of HSA-bFGF NPs slightly increased from 167 to 200 nm, this degree of size change can be interpreted as natural swelling due to long-term incubation in the solution [50, 51]. In addition, the morphology of 14-day-old HSA-bFGF NPs was observed through SEM in Fig. S1B. There was no significant difference in the shape of the 2-week-old protein nanoparticles, compared to the morphology of the nanoparticles immediately generated (Fig. 2H). Therefore, the HSA-bFGF NPs produced in this study are considered to be very stable, and thus are considered very suitable as a platform for releasing and providing intact bFGF protein.

We then assessed the bFGF release profile under in vitro physiological condition. The bFGF was readily released from the protein nanoparticles with sustained release kinetics while showing an incremental release rate with the lower crosslinking efficiency (Fig. 2I). Upon crosslinking with a higher amount of GA, the HSA and bFGF proteins construct a higher degree of networking and assembly, resulting in slower release profile. Therefore, the bFGF release can be easily modulated while maintaining other properties (size, PI, and zeta-potential) by adjusting the volume of GA crosslinker added during the nanoparticle formation process. Considering the initial amount of bFGF added to generate the HSA-bFGF NPs, we calculated the percentage of the released bFGF protein. And then we represented released profile of bFGF from the HSA-bFGF NPs, reflecting the percentage in Fig. S1C. According to the results, it was confirmed that intact bFGF protein was accumulated and released up to 8% for a total of 12 days. And bFGF protein was observed to be continuously released from the HSA-bFGF NPs at about 1% per day, when crosslinked with 1.5 µl of 10% GA. Since we used 20 µg/ml of HSA-bFGF NPs (containing 5 µg/ml of bFGF protein and crosslinked with 1.5 µl of 10% GA), it is thought that 40–50 ng/ml of bFGF protein, corresponding to 1%, was supplied daily to cells or tissues, which is sufficient concentration for regeneration efficacy.

### Enhanced cell proliferation and migration with HSA-bFGF NPs

**Fig. 3:**
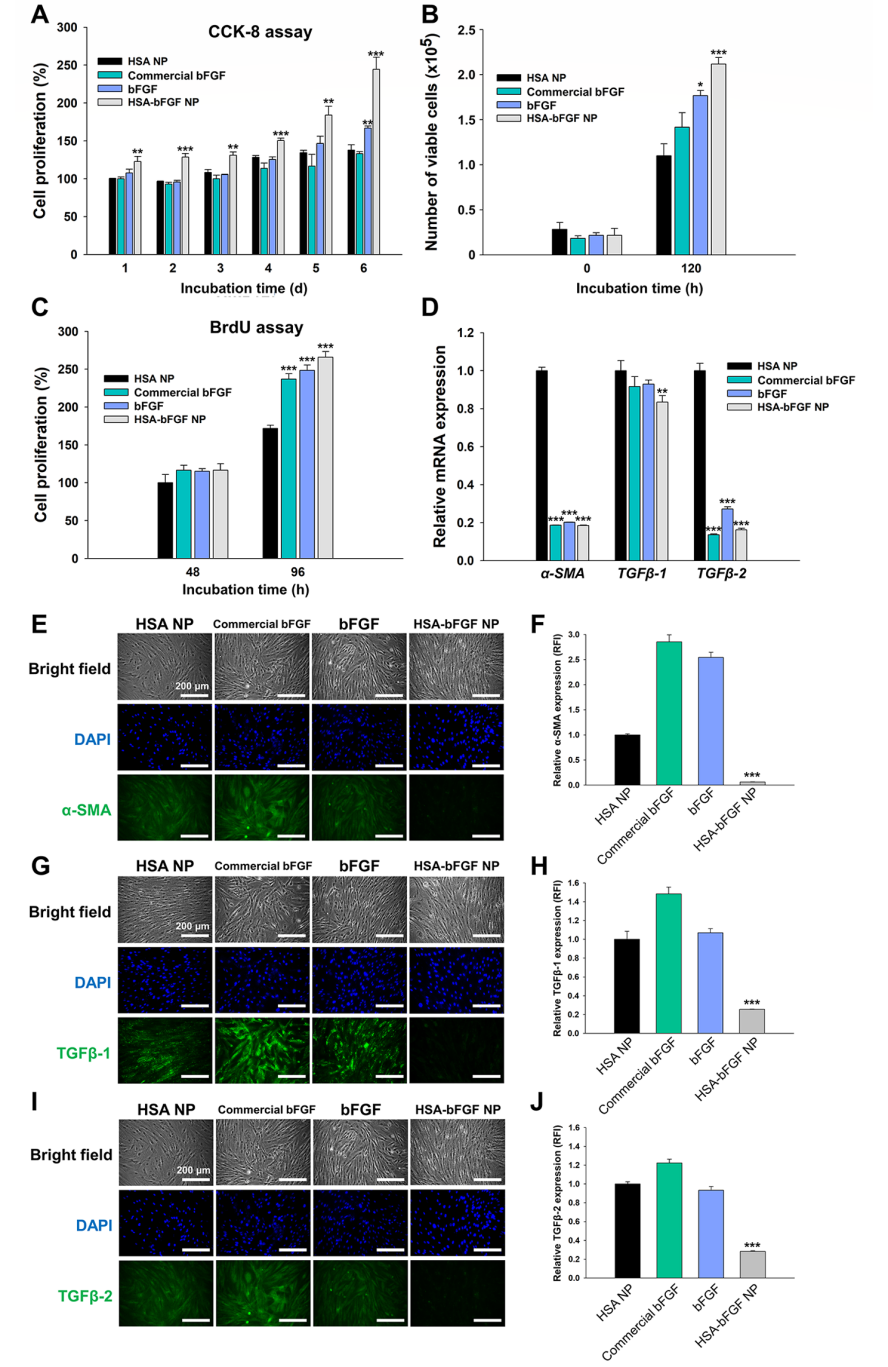
Cell proliferation activity and anti-fibrosis efficiency of HSA-bFGF NPs in vitro. (**A**) CCK-8 assay of HDFs for cell proliferation analysis. (**B**) Cell counting assay of HDFs for measuring number of viable cells. (**C**) BrdU assay of HDFs for DNA synthesis analysis. (**D**) Relative mRNA expression of myofibroblast-related genes represented as *α-SMA*, *TGFβ-1*, and *TGFβ-2*. (**E, G, I**) Immunocytochemical analysis of fibrosis marker proteins. Nuclei were represented as blue, and fibrosis marker proteins, α-SMA (**E**), TGFβ-1 (**G**), and TGFβ-2 (**I**), were shown as green, respectively. Scale bars, 200 μm. (**F, H, J**) Quantified fluorescence intensity of fibrosis marker proteins, respectively. * *p* < 0.05, ** *p* < 0.01 and *** *p* < 0.001, compared to HSA NP as a control. *n = 3*

The HSA-bFGF NPs were further investigated to verify whether the cell proliferation and migration were affected by sustained supply of bFGF. Human dermal fibroblasts (HDFs) treated with HSA-bFGF NPs showed enhanced proliferation compared with cells treated with HSA NPs without bFGF, commercially purchased bFGF protein (commercial bFGF), and produced bFGF protein (bFGF) (Fig. 3A and B). After 5 days of treatments, HDF proliferation rate significantly increased by more than 50% in cells with HSA-bFGF NPs, compared to cells with HSA NPs or commercial bFGF (Fig. 3A), resulting in statistically significant increase in viable cell number (Fig. 3B). Thus, sustained release of bFGF should be responsible for the enhanced proliferation of HDFs. Cell proliferation was also confirmed by the 5-bromo-2′-deoxyuridine (BrdU) assay quantifying deoxyribonucleic acid (DNA) synthesis, showing incremental DNA synthesis facilitated with commercial bFGF, bFGF, and HSA-bFGF NPs on day 4 (Fig. 3C).

Since the permanent differentiation of the fibroblasts to myofibroblasts is one of the key mediators for scar formation in wound healing process, the effect of bFGF proteins on scarless skin regeneration was also investigated. To confirm the inhibitory effect in the fibroblast differentiation by HSA-bFGF NPs, expression of myofibroblast-associated marker genes, including *α-smooth muscle actin* (*α-SMA*), *transforming growth factor β-1* (*TGFβ-1*), and *transforming growth factor β-2* (*TGFβ-2*) was observed (Fig. 3D). Cells were harvested after 2 days of treatment with HSA NPs, commercial bFGF, bFGF or HSA-bFGF NPs for quantified reverse transcriptase-polymerase chain reaction (qRT-PCR) analysis. The expression of both *α-SMA* and *TGFβ-2* was 80% down-regulated upon bFGF treatment regardless of the delivery form (soluble protein or protein nanoparticles). The *TGFβ-1* expression level was not significantly down-regulated in cells with commercial bFGF or bFGF compared to HSA NPs-treated cells, while mRNA level of *TGFβ-1* in cells with HSA-bFGF NPs showed statistical decrease compared with the only HSA NPs. Furthermore, those scar formation-related markers were investigated in protein level (Fig. 3E to J). According to the immunocytochemical analysis of α-SMA (Fig. 3E), TGFβ-1 (Fig. 3G), TGFβ-2 (Fig. 3I), protein expression level obviously decreased in HSA-bFGF NPs-treated cells compared to the other groups. And the quantified fluorescence intensity indicates that myofibroblast-associated proteins statistically decreased in cells with HSA-bFGF NPs, compared with not only the HSA NPs but also soluble bFGF protein (both commercial bFGF and bFGF) (Fig. 3F, H, J).

**Fig. 4:**
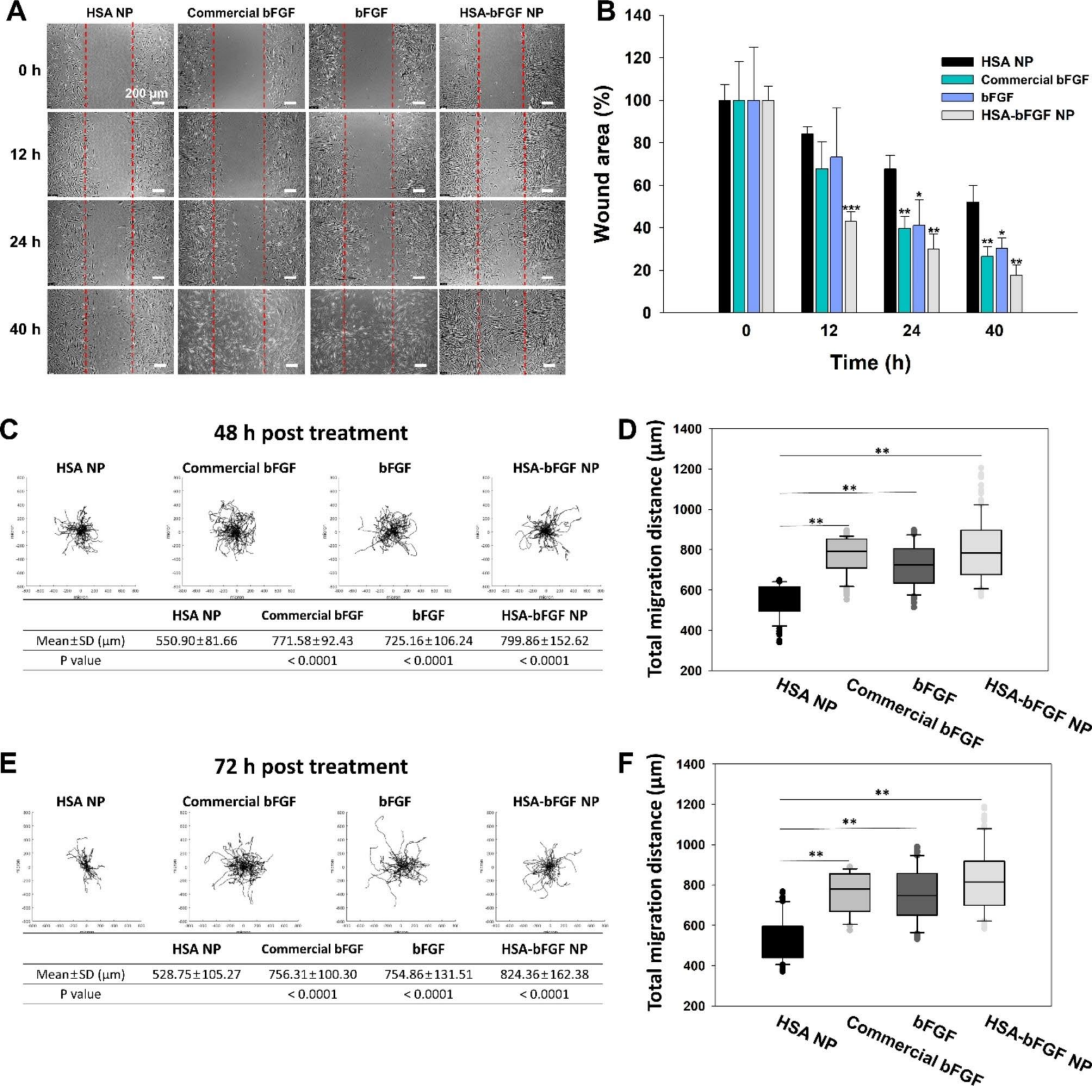
Cell migration activity of HSA-bFGF NPs in vitro. (**A**) Wound area closure assay. Migration of HDFs was observed through a microscope depending upon the incubation time (0 to 40 h), when treated with HSA NP (as a control), commercial bFGF, bFGF, and HSA-bFGF NPs, respectively. Scale bars, 200 μm. (**B**) Quantified values of wound area. * *p* < 0.05, ** *p* < 0.01 and *** *p* < 0.001, compared with each control (HSA NP group at the same time point). *n = 3*. (**C, D, E, F**) Single cell trajectory analysis. (**C**) Trajectories of a HDF after 48 h of protein or protein nanoparticle treatment. (**D**) Quantified total migration distance of the HDFs after 48 h. (**E**) Trajectories of a HDF after 72 h of protein or protein nanoparticle treatment. (**F**) Quantified total migration distance of the HDFs after 72 h

Corresponding to the proliferation of HDFs, the HSA-bFGF NPs also contributed to accelerated migration of the cells in wound closure test in vitro (Fig. 4A and B). According to the microscopic images in Fig. 4A, the HDFs treated with HSA-bFGF NPs showed enhanced migration activity, compared to the other groups including HSA NPs-treated cells and soluble bFGF protein-treated cells. After 12 h of treatments, the wound area of HDFs showed obviously rapid recovery with HSA-bFGF NPs compared with other groups (Fig. 4B). The wound area of HDFs was observed to significantly diminish in HDFs treated with the HSA-bFGF NPs at extended time, leading to 80% of wound closure in 40 h, whereas the HSA NPs-treated HDFs showed 50% of closure in wound area.

Next, we evaluated the persistence of bFGF affecting the cell migration by single cell tracking analysis (Fig. 4C to F). Representative cell trajectories, observed at 48 h after the protein or protein nanoparticles treatment, showed that total migration distance of the cells treated with HSA-bFGF NPs increased by 45% compared to the control with HSA NPs (Fig. 4C and D). In addition, after 72 h of treatment, the total migration distance of the HDFs increased even more, approximately 56%, by the HSA-bFGF NPs, compared with HSA NPs (Fig. 4E and F). Moreover, as the time was further extended from 48 to 72 h, enhanced migration activity by HSA-bFGF NPs was remarkable compared to not only HSA NPs but also soluble bFGF protein. Resultingly, the effect of bFGF protein as soluble form (commercial bFGF and bFGF) on migration activation was rapidly saturated due to short half-life of the protein, while the bFGF released from the protein nanoparticles could show continuous and accumulated efficacy.

### In vivo wound healing and tissue regeneration

**Fig. 5:**
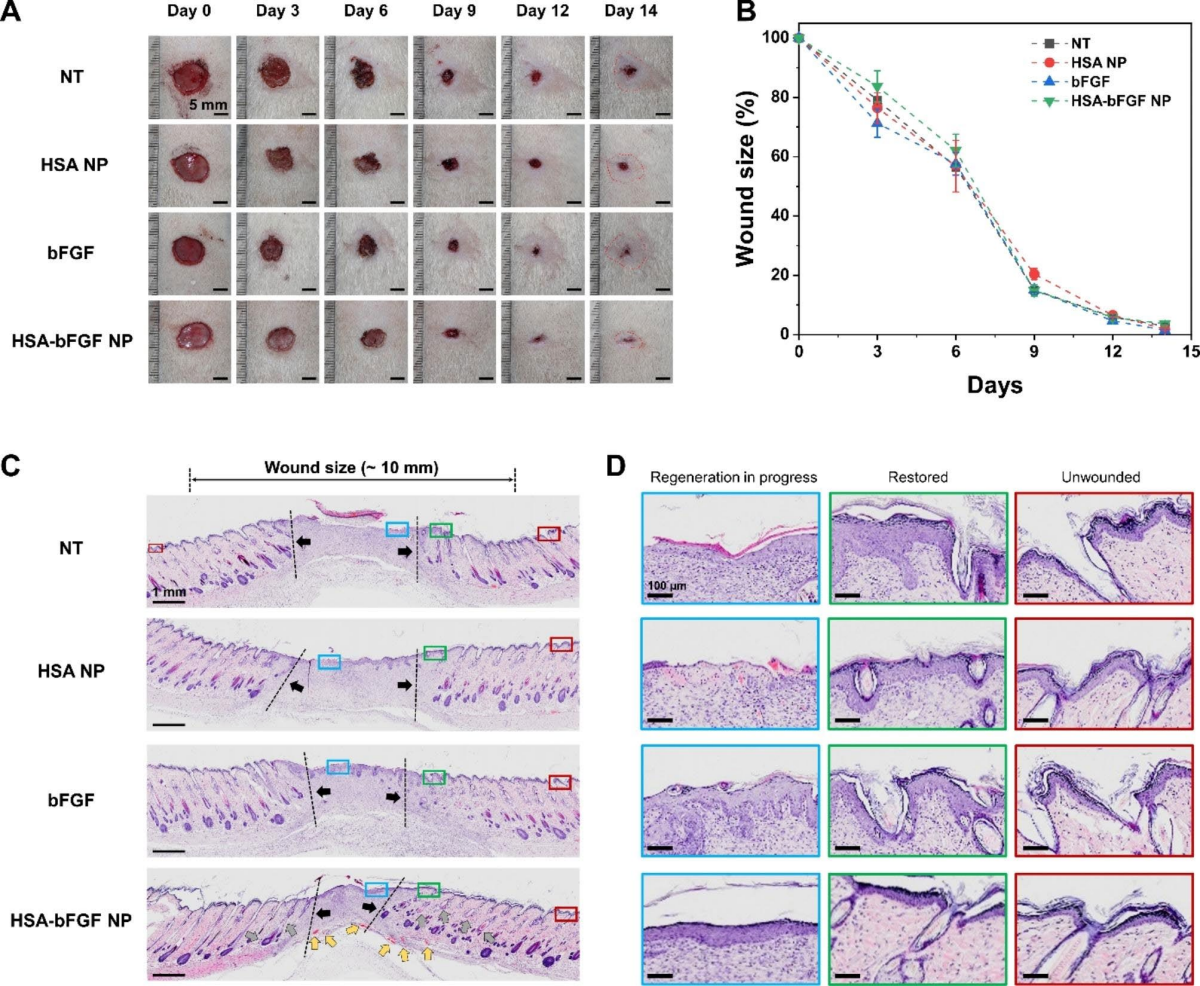
In vivo wound healing activity and histological evaluation of wound sections. (**A**) Representative photographs of the rat skin wound healing treated with HSA NPs, soluble bFGF, and HSA-bFGF NPs, compared to NT at day 0, 3, 6, 9, 12, and 14. Dosage of soluble bFGF was 12.5 µg, and equivalent extent of bFGF was loaded in HSA-bFGF NPs. The boundary between the hairy part and the non-hairy part of the skin was marked (dashed line, red). Scale bars, 5 mm. See enlarged images in Fig. S3. (**B**) Quantitatively analyzed wound size reduction profile. *n = 3 to 5*. (**C**) Representative H&E staining images of the wound skin upon different treatments. Panniculus gap was presented as dashed lines and arrows (black). HSA-bFGF NPs showed significant tissue recovery with a large number of blood vessels (yellow arrows) and hair follicles (gray arrows) compared to other groups. Scale bars, 1 mm. See enlarged images in Fig. S4. (**D**) Magnified H&E staining images of each skin designated as solid boxes in (**C**) showing differences in tissue microstructures after wound healing process upon each treatment. Regeneration in progress was exhibited in blue box, restored region was in green box, and unwounded area was in red box, respectively. Stratum corneum and thickness of epidermis layer was formed similar to healthy skin in treated with HSA-bFGF NPs. Capillaries and glands were also found to be more comparable to intact epidermal structure. Scale bars, 100 μm

The HSA-bFGF NPs were further investigated to evaluate the curative effect on skin regeneration in wound healing model of rat in vivo. Excisional skin wound (~ 1 cm in diameter) was prepared on the dorsum of specific pathogen free (SPF) Sprague Dawley (SD) rats, and those rats were divided into four groups: non-treated rats as a control (NT) and experimental rats treated with HSA NPs, soluble bFGF, and HSA-bFGF NPs, respectively. In Fig. 5, the bFGF dosage was fixed to be 12.5 µg. The results showed that soluble bFGF-treated skin showed slightly better wound closure kinetics than other groups, possibly due to beneficial effect of higher bFGF supplement at the initial stage of wound healing (Fig. 5A and B). However, the curative effect of bFGF is hardly maintained over time because of short half-life, resulting in statistically non-significant improvement of wound closure. On the other hand, sustained release of intact bFGF from HSA-bFGF NPs supports substantially higher efficacy in skin regeneration, as the activity of bFGF lasts longer than soluble bFGF treatment. In macroscopic evaluation, the hairy area at wound site was considerably larger with the HSA-bFGF NPs treatment than other groups (Fig. 5A and Fig. S3). Although the wound closure seemed to be comparable in size after treatment with bFGF and HSA-bFGF NPs, most area of the regenerated skin showed hair growth recovery with HSA-bFGF NPs treatment, while bFGF-treated mice still showed limited hairy area. The results emphasize that the bFGF is beneficial for earlier wound closure at initial stage but long-term sustained release of bFGF as an active form from HSA-bFGF NPs is even more important for improved tissue regeneration with its own function in the aspect of hair growth.

Tissue regeneration and microstructural remodeling processes are critical phases that determine the quality of the recovered skin [9], thus we further assessed histological investigation of the regenerated skin (Fig. 5C and D). Despite of partial appearance of a few collagen fibers at day 14, natural wound healing process formed the fibrous collagen with loose and disordered structure in the non-treated group. However, the HSA-bFGF NPs-treated group showed highest level of collagen deposition with the dense and thick collagen fibers of better arrangement in skin tissues because of much stable differentiation and restoration upon sustained release of bFGF from the HSA-bFGF NPs, compared to the other groups. Moreover, large number of blood vessels and hair follicles with capillaries and glands were also found in the regenerated dermal tissues treated with HSA-bFGF NPs (Fig. 5D and Fig. S4). Particularly, the blood vessels were well regenerated with HSA-bFGF NPs in the restored region, which was comparable to the unwounded (normal) skin, while other groups showed the thicker epithelial layer and very low evidence of blood vessel formation in the restored region. It should be noted that excessive epithelial differentiation and thicker epithelial layer obtained with bFGF treatment potentially indicate an abnormal pattern of tissue regeneration, with a higher likelihood of scar formation [52–54]. In the cutaneous wound healing, balanced tissue regeneration process as a course of hemostasis, inflammation, proliferation, and remodeling is crucial to avoid scar formation. In this regard, excessive bFGF at early stage of regeneration might be causing rapid epithelial proliferation and differentiation followed by thicker epithelium layer formation, which potentially causes adverse effects on proper tissue regeneration. Sustained release of bFGF from the HSA-bFGF NPs, however, provides a suitable environment for better recovery of skin in terms of function as it restores more blood vessels and hair follicles in short period of time. Overall, the long-term sustained release of bFGF with its intrinsic activity using HSA-associated nanoparticle formulation (HSA-bFGF NPs) showed significant improvement for better skin regeneration than one-shot burst supplement of bFGF as it is deactivated rapidly in physiological condition.

**Fig. 6:**
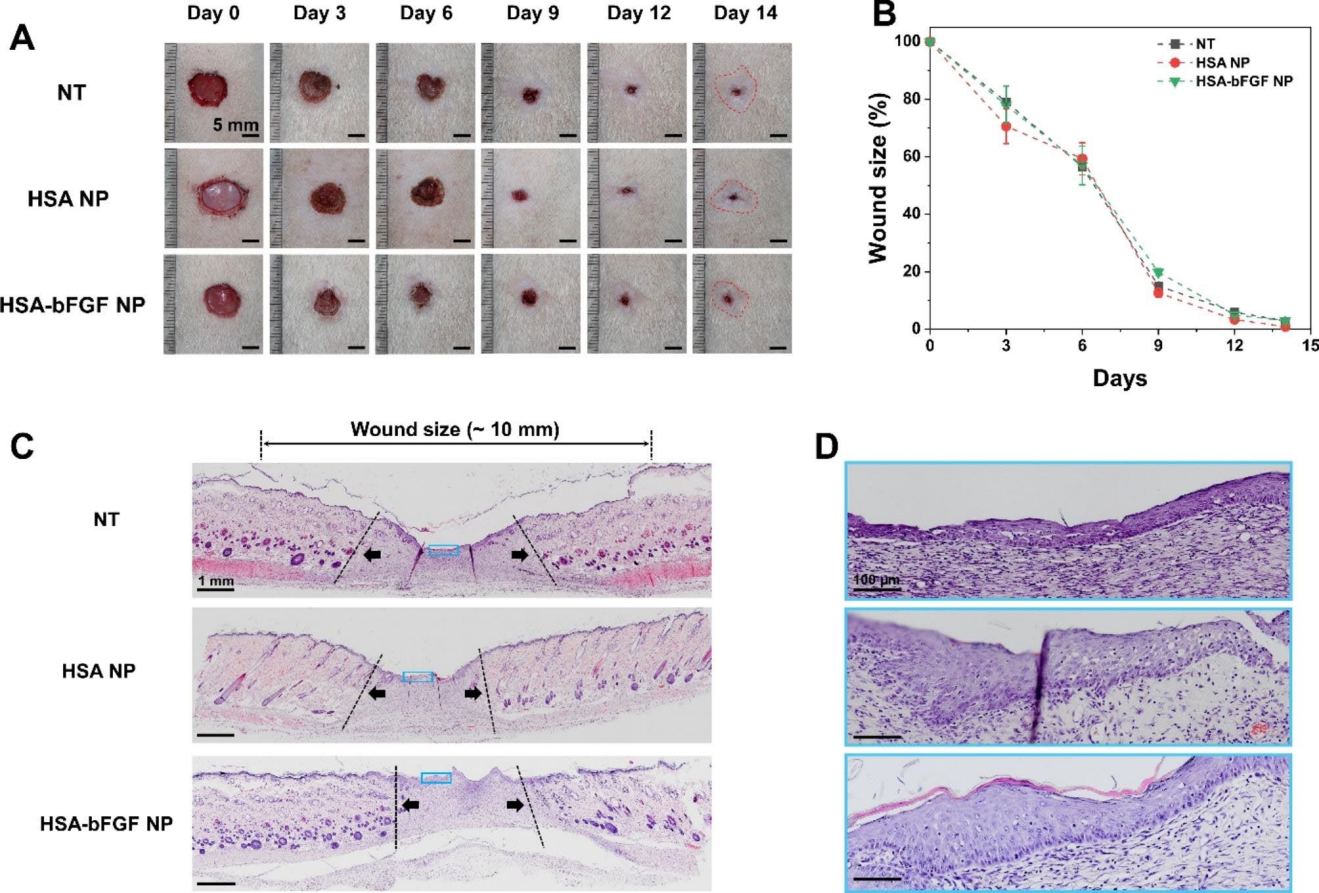
Low dose wound healing activity and histological evaluation of wound sections in vivo. (**A**) Representative photographs of the rat skin wound healing treated with HSA NPs and HSA-bFGF NPs, compared to NT at day 0, 3, 6, 9, 12, and 14. Dosage of loaded bFGF in HSA-bFGF NP was 3 µg. The boundary between hairy area and the non-hairy area of skin was marked (dashed line, red). Scale bars, 5 mm. (**B**) Quantitatively analyzed wound size reduction profile. *n = 3 to 5*. (**C**) Representative H&E staining images of the wound skin upon different treatments. Panniculus gap was presented as dashed lines and arrows (black). Thickness of regenerated skin treated with HSA-bFGF NPs was thicker compared to other groups. Scale bars, 1 mm. (**D**) Magnified H&E staining images of each skin designated as a solid box (blue) in (**C**). Though hyperproliferative epidermis layer shows not completely recover the wound, the wound was shown to be in the process of recovering. Scale bars, 100 μm

We also investigated the curative effect of HSA-bFGF NPs at 4-fold lower dosage of bFGF (3 µg) to demonstrate dose-dependent efficacy in tissue regeneration (Fig. 6). The wound closure rate of HSA-bFGF showed no significant difference with other groups (Fig. 6A and B). In addition, regenerated tissues were slightly thicker with 3 µg of HSA-bFGF NPs treatment compared with higher dosage (12.5 µg) HSA-bFGF NPs treatment (Figs. 5D and 6D). Since the thicker epidermal layer formed in the lower dose treatment is likely to cause scarring as noted above, lower dosage of HSA-bFGF NPs has limited efficacy to enhance the tissue regeneration [55]. Correspondingly, it is evident that the HSA-bFGF NPs at a 4-fold lower dosage (3 µg) is less sufficient to achieve tissue recovery compared to the higher dosage (12.5 µg) treatment. While sustained supplement of bFGF is crucial for recovering not only anatomical tissue structure but also its function, in vivo studies also support that properly sufficient extent of bFGF is required to completely cure the excisional skin wound.

**Fig. 7:**
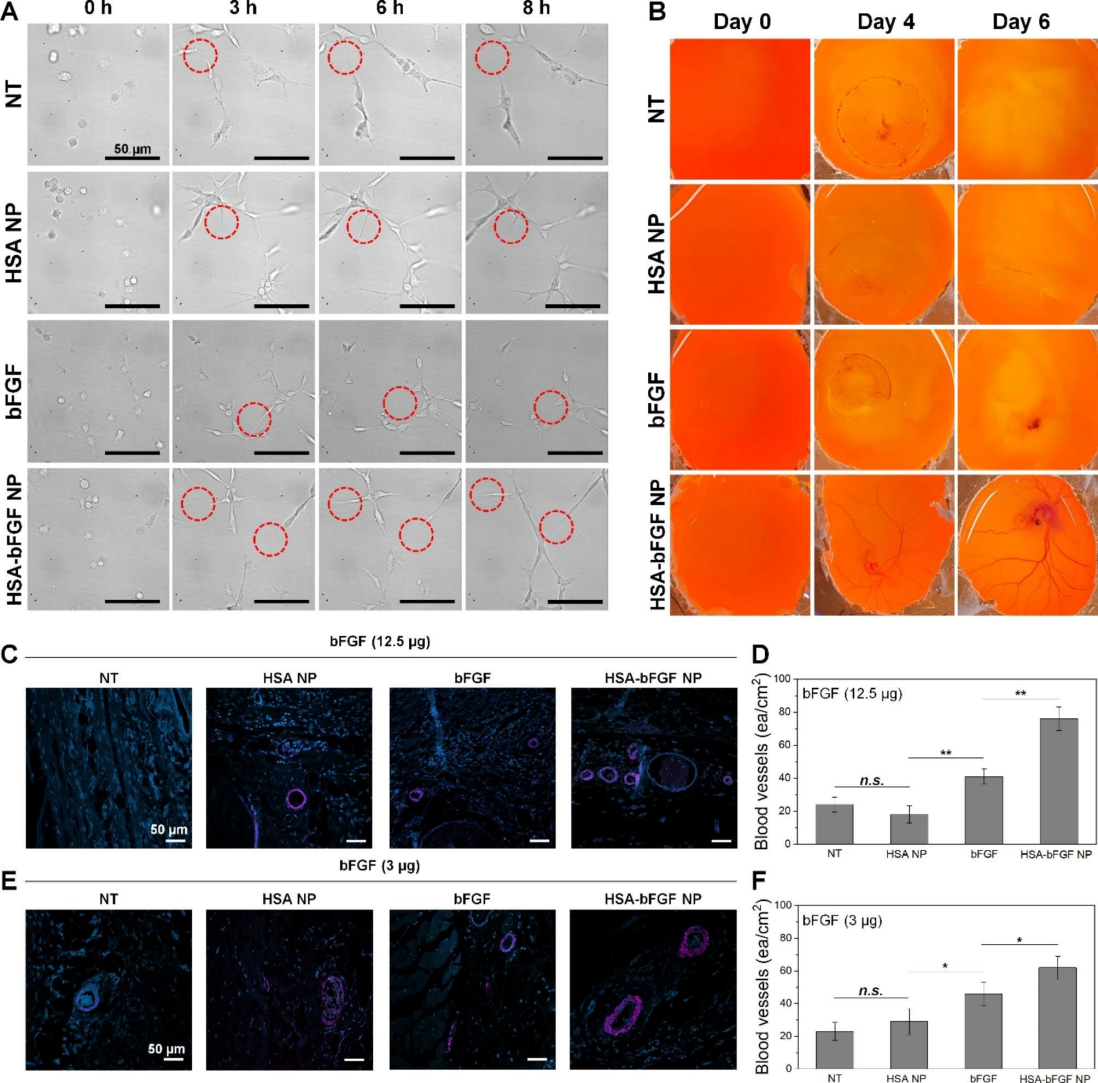
Angiogenic activity of HSA-bFGF NPs in vitro and in vivo. (**A**) Observation of angiogenic tube formation process of the HUVECs. Images showed sprouting from the HUVECs as indicated (red dotted circles). The HSA-bFGF NPs-treated cells maintained sprouting activity until 8 h. Scale bars, 50 μm. (**B**) In vivo angiogenesis evaluation through CAM assay. Extended blood vessel formation in fertilized eggs was observed upon treatment with HSA-bFGF NPs, compared with the other groups. Photographic images were taken at day 0, 4 and 6. (**C, E**) Immunofluorescence analysis of angiogenesis in the skin wound in vivo. Angiogenic efficacy was validated through observation of blood vessel formation in wounded area of rats. Blood vessels (magenta) and nuclei (cyan) in the dermis and hypodermis were stained with fluorescence labelled CD31 antibody and Hoechst 33342, respectively. Wound was treated with HSA NPs, soluble bFGF, and HSA-bFGF NPs at the bFGF contents of (**C**) 12.5 µg and (**E**) 3 µg, respectively. Scale bar, 50 μm. (**D, F**) Quantitative analysis of blood vessel numbers in wound area. HSA-bFGF NPs showed superior healing capability over any other groups, in accordance with macroscopic analysis. *n.s.* not significant. * *p* < 0.05 and ** *p* < 0.01. *n =* 3

To further evaluate the efficacy of HSA-bFGF NPs on tissue regeneration, we investigated the curative effect in terms of angiogenesis (Fig. 7). As discussed above, the bFGF has been known to promote neovascularization in damaged skin tissue, aiding in wound healing [56–59]. In this regard, enhanced angiogenesis was determined at cellular level by tracking angiogenic tube formation (Fig. 7A). Human umbilical vein endothelial cells (HUVECs) were seeded onto extracellular matrix (ECM)-like gel and allowed to form connections with neighboring cells. We observed rapid progression of angiogenesis evidenced by stable interconnection of the cells treated with HSA-bFGF NPs. While other groups showed unstable connections with neighboring cells and hardly progressed in terms of angiogenesis. Until 8 h, the HSA-bFGF NPs treatment maintained intercellular connections, which is potentially correlated with sustained blood vessel formation as endothelial cells organize themselves and begin to extend, followed by aligning into tubular structures resembling blood vessels. Moreover, the HSA-bFGF NPs-treated cells exhibited remarkably noticeable stability with higher number of the nodes responsible for intercellular connection at extended period of culture, possibly due to sustained supplement of bFGF (Fig. S5). While the number of branches for blood vessel formation was comparable in each group, the higher degree of nodes supports feasible physiological environment for the endothelial cells in culture media.

Also, chorioallantoic membrane (CAM) assay was conducted using fertilized eggs. We observed that the groups treated with NT, HSA, and soluble bFGF did not show blood vessel formation within 6 days after incubation. However, upon treatment with HSA-bFGF NPs, significantly higher number of blood vessels were started to form at day 4 (Fig. 7B).

Furthermore, in vivo immunofluorescence staining was conducted using an endothelial cell marker CD31 (magenta) to visualize the blood vessel formation. As a result, higher angiogenesis was observed with HSA-bFGF NPs treatment, in accordance with histological analysis (Fig. 7C and E). Significantly higher number of blood vessel found in the recovered tissue indicates that the gradual supplement of bFGF is crucial for improved skin regeneration rather than burst administration of bFGF (Fig. 7D and F). Therefore, secured loading of bFGF as a particulate form with HSA is markedly beneficial for long-term stability of bFGF, followed by retained activity for tissue regeneration and blood vessel formation. The results indicated the essential role of bFGF in efficient skin tissue regeneration despite of its short half-life. Overall, we confirmed not only the contribution of bFGF on skin regeneration but also the importance of intact activity of bFGF to maintain the curative effect for complete recovery over time.

## Discussion

Since the bFGF is involved in many biological responses including collagen deposition and angiogenesis during wound healing process, bFGF has been studied for a number of therapeutic uses in the field of regenerative medicine [56–58]. Moreover, the bFGF protein possesses intrinsic properties such as improving cell viability [59, 60], proliferation [61, 62], and migration [10, 63, 64], which have been considered as an essential component for tissue regeneration. However, there have been limitations in clinical applications of the bFGF protein because the large-scale production of bFGF requires complicated and laborious steps [17–19] and the half-life of the bFGF was quite short (~ 8 h) in physiological conditions [20, 21].

In this study, to overcome those drawbacks of bFGF usage, soluble expression and mass production of the bFGF protein was readily facilitated by using the mutated vector [49] (Fig. 2A to D). Next, to enhance the stability of bFGF protein, HSA NPs were manufactured to contain bFGF payloads, resulting in stable formulation of HSA-bFGF NPs (Fig. 2E to I). We previously reported that, in the form of HSA NPs, the stability of cargo proteins is significantly enhanced under both moderate and harsh conditions in vitro [65–68]. As a drug delivery vehicle, the HSA NPs could stabilize the activity of cargo not only during the preparation process but also in the intracellular environment after delivery to the cells [69].

Here, the HSA-bFGF NPs were generated through a desolvation method, where aqueous proteins agglomerate together to form spherical nanoparticles with homogeneous size distribution, upon adding drops of ethanol. Then the protein aggregates were stabilized using GA, a crosslinking agent. The HSA-bFGF NPs conserved their size, PI, zeta-potential, and morphology regardless of the bFGF contents ratio (0 to 25%) in HSA-bFGF NPs (Fig. 2F to H). In the form of protein nanoparticles, bFGF was consistently released into media for 12 d, and the release of bFGF could be easily modulated by regulating the addition of crosslinker (Fig. 2I). According to Fig. S1C, bFGF protein was continuously released from the HSA-bFGF NPs at about 1% per day, which means that 40–50 ng/ml of bFGF protein was supplied daily. According to the related studies using the bFGF for the purpose of maintaining the pluripotency in stem cells such as embryonic stem cells (ESCs) and induced pluripotent stem cells (iPSCs) [70, 71], or for the purpose of tissue regeneration with enhanced angiogenesis [72, 73], 10 ng/ml has been widely used. Thus, 40–50 ng/ml (1% per day) would be a sufficient concentration for regeneration efficacy.

According to the results, HSA-bFGF NPs showed significant effects on increased cell proliferation (Fig. 3A, B and C), decreased risk of scar formation (Fig. 3D to J), and enhanced cell migration (Fig. 4) through in vitro validations. However, wound closure rate showed negligible enhancement with HSA-bFGF NPs compared to soluble bFGF protein treatment in vivo (Figs. 5 and 6 A). Although the beneficial contribution of bFGF protein for dermal cell proliferation and migration during wound healing process is obviously demonstrated in vitro, the concentration of bFGF released from HSA-bFGF NPs at early stage of wound might not be sufficient to improve the skin regeneration in vivo. On the other hand, the results indicate that soluble bFGF is much susceptible to physiological condition, thus readily inactivated, resulting in less effective tissue regeneration. While burst supplement of bFGF leads slightly rapid wound closure in a limited period of time at early stage (within 3 days) of wound in vivo, the HSA-bFGF NPs gradually release the active form of intact bFGF for much extended time, leading improved microscopic tissue regeneration to recover the intact function (e.g., less scar formation, larger hairy area, etc.) Overall, considering the sustained release of bFGF payloads from the HSA-bFGF NPs, secure protection, and long-term supply of bFGF could lead substantially enhanced wound healing in terms of dermal tissue regeneration and functionality, as indicated in recovered hairy area at the wound site (Fig. 5).

Regarding the histological evaluation, collagen was dramatically reduced in the damaged skin tissue, which leads poor wound healing and impaired tissue remodeling (Figs. 5 and 6). The histological investigations indicated that the HSA-bFGF NPs had the thicker granulation tissue width than other groups. Thus, the bFGF-induced accelerated skin regeneration was particularly related with enhanced angiogenesis. In addition, the skin tissue was regenerated in the form of a native tissue-resembling structure with a significantly decreased panniculus gap and newly formed appendages. Since the wound crust (scab) appeared to cover the wound area in the early stage, rapid scab formation and complete epidermal layer regeneration are responsible for enhanced re-epithelialization process (Fig. 7). Broad distribution of newly formed blood vessels induced by sustained release of bFGF during the regeneration process is mostly responsible for complete recovery of excisional wound. According to the results, soluble bFGF dose over 10 µg was shown to be beneficially effective in tissue regeneration, as a means of enhanced blood vessel formation and strengthened ECM. Moreover, according to the CAM assay (Fig. 7B), increased stability and sustained release of bFGF by forming HSA-bFGF NPs contributed to the prolonged efficacy of bFGF, thereby demonstrating beneficial effect on angiogenesis in vivo. Although the microscopic observation of angiogenesis is hardly taken during the course of skin regeneration in rat model in vivo, an alternative model of in vivo angiogenesis using CAM assay supports our findings on improved tissue regeneration in terms of stable supplements of bFGF. Therefore, the present study strongly supports that the HSA-bFGF NPs had a critical contribution to better quality of tissue regeneration led by efficient re-epithelialization and angiogenesis with long-term sustained release of bFGF in vivo.

## Conclusion

We utilized the HSA in the form of protein nanoparticles to securely load the bFGF proteins. The HSA-bFGF NPs have a spherical shape and uniform size distribution while maintaining the intact activity of bFGF payloads. The bFGF proteins were released from the HSA-bFGF NPs in a sustained manner, and the bFGF release kinetics were readily modulated upon controlling the amount of crosslinker. The HSA-bFGF NPs also exhibited enhanced cell proliferation, improved cell migration, and down-regulated risk of scar formation activity with low cytotoxicity, compared with soluble bFGF (both commercially purchased and produced followed by purification), and HSA NPs without bFGF protein in vitro. We have also shown that HSA-bFGF NPs conserved intact properties of the bFGF protein, improving tissue regeneration, especially in microstructural and functional recovery in vivo. Furthermore, enhanced angiogenic activity of the HSA-bFGF NPs were thoroughly investigated through in vitro, CAM, and in vivo analysis. Therefore, HSA-bFGF NPs are strongly expected to be used as an efficient tool for the delivery of therapeutic protein, bFGF, in order to cure wounded tissues or organs in the field of regenerative medicine. Furthermore, not limited to the bFGF protein, the HSA-based protein nanoparticles would be a useful formulation for in vivo protein delivery by facilitating secure loading and controllable release profile.

## Methods

### Production of bFGF protein

The bFGF protein was produced using a pET-28a expression vector (Novagen, WI, USA) containing bFGF-G3 gene with a 6 × His-tag and Agg thrombin-tag at the N-terminus. After transformation with a pET-28a/*FGF2-G3* vector, *E. coli* BL21 (Novagen) was cultured in LB medium, containing 100 µg/ml ampicillin at 37 ℃. Then the cells were induced with 1 mM of IPTG (Calbiochem, USA), followed by further incubation at 37 ℃ for 4 h or at 30 ℃ for 16 h. After harvesting, cells were disrupted by ultrasonication and then centrifuged. The bFGF protein was purified from the supernatant using a HisTrap HP column (Cytiva, USA) and dialyzed with Tris-HCl buffer (20 mM Tris-HCl, pH 8.0) using a desalting column (Cytiva). The purified bFGF protein was stored at -20 ℃ before use. The amount of the bFGF protein was analyzed using the software for quantification, Gen5 (BioTek, USA). Different from the produced bFGF protein (bFGF), commercially purchased bFGF protein (commercial bFGF, Peprotech, Korea) was also used as another control group.

### SDS-PAGE and immunoblotting

To confirm the size and quantify the amounts of bFGF proteins, SDS-PAGE was performed. Total cell lysate, soluble fraction, and the purified bFGF protein were respectively mixed with reducing sample buffer containing SDS and β-mercaptoethanol (pH 6.8), prior to loading on a 12% SDS-PAGE gel. Through the electrophoresis, each sample was separated depending upon size. Then, the polyacrylamide gel was immersed in Coomassie blue staining solution for visualization. In order to identify the bFGF proteins, Western blot analysis was conducted. After transfer of total cell lysate, soluble fraction, and the purified bFGF products respectively on SDS-PAGE gel to polyvinylidene difluoride (PVDF) membrane, proteins were labeled with anti-bFGF antibody (Abcam, UK). Clarity Western enhanced chemiluminescence (ECL) substrate with horseradish peroxidase (HRP)-conjugated secondary antibody (Bio-Rad, CA, USA) was then used to visualize proteins on the G-Box Chemi XL system (Syngene, UK).

### Preparation of protein nanoparticles

The HSA-bFGF protein nanoparticles (HSA-bFGF NPs) were prepared using a desolvation method. The 1 mg of HSA (Sigma Aldrich, MO, USA), and the various amounts of purified bFGF protein were dissolved in 1 ml Tris-HCl buffer (pH 8.0) under constant stirring (500 rpm) at room temperature. Then, 4 ml of 100% ethanol was added drop wise using a syringe pump under constant agitation in order to form nano-sized protein aggregates. After desolvation, the HSA-bFGF NPs were crosslinked by addition of 10% GA aqueous solution (0.5 to 5 µl), and then they were stirred for 1 h at room temperature. The protein nanoparticles were purified by centrifugation at 12,000 ×g at 4 ℃ for 15 min. After discarding the supernatant, pellets of protein nanoparticles were resuspended in PBS.

### Size, PI and zeta-potential of the protein nanoparticles

The size, PI and zeta-potential of the prepared nanoparticles were determined with an ELS spectrophotometer (ELS-8000, Otsuka, Tokyo, Japan). The HSA-bFGF NPs were centrifuged and resuspended in DIW before investigation. The size, PI, and zeta-potential of the protein nanoparticles were determined using the corresponding mode of the ELS spectrometer according to the manufacturer’s instructions.

### SEM analysis of the protein nanoparticles

In order to investigate the morphological characteristics of the protein nanoparticles, SEM analysis was performed. After diluted to 1/10 with distilled water (DW), droplets of HSA-bFGF NPs aqueous solution were placed on a 12 mm circular coverslip, lyophilized using a freeze-dryer (Hanil, Seoul, Korea), and then sputter-coated with the platinum. The SEM images were obtained using a field emission SEM (FE-SEM; JSM-6700 F, JEOL, Japan).

### In vitro release of bFGF protein from the protein nanoparticles

To assess in vitro bFGF release from the HSA-bFGF NPs, enzyme-linked immunosorbent assay (ELISA) was performed. Every day for 12 d at 37 ℃, each supernatant was separated using the centrifugation from the incubated protein nanoparticles with different amount of the crosslinker, GA. For ELISA, the prepared samples were diluted 1/1,000, and the absorbance was measured using an ELISA kit (BioLegend, CA, USA) through Tecan.

### Analysis of cell proliferation

The proliferation of HDFs was assessed using cell counting kit-8 (CCK-8) kit (Dojindo Molecular Technologies, USA). After HDFs were seeded in 96-well plates and incubated for 24 h, cells were treated with 20 µg/ml of control HSA NPs, 5 µg/ml of commercial bFGF, 5 µg/ml of bFGF, and 20 µg/ml of HSA-bFGF NPs, respectively. Then CCK-8 assay was performed for 6 d. To quantify the number of live cells possessing mitochondrial activity, CCK-8 reagent (1 µl/ml, diluted in culture media) was added to each well and incubated for 90 min. Then the optical density (OD) value was measured using a microplate reader (BioTek™ Eon™ Microplate Spectrophotometers, USA) at 450 nm. Also, number of viable cells were calculated by counting cells after treatment of bFGF protein or protein nanoparticles. After HDFs were seeded in 24-well plates, cells were incubated with 20 µg/ml of control HSA NPs, 5 µg/ml of commercial bFGF, 5 µg/ml of bFGF, and 20 µg/ml of HSA-bFGF NPs, respectively for 0 or 120 h. After trypan blue staining, unstained viable cells were counted through hemocytometer.

### Quantification of DNA synthesis

To quantify the DNA synthesis of HDFs under the influence of HSA-bFGF NPs, BrdU assay kit (Cell Signaling Technology, USA) was used. When cells were cultured with BrdU-containing media, the pyrimidine, a type of nucleotide analog, incorporated into DNA replacing thymidine. After HDFs were seeded onto 48-well plate, 20 µg/ml of control HSA NPs, 5 µg/ml of commercial bFGF, 5 µg/ml of bFGF, and 20 µg/ml of HSA-bFGF NPs were treated to the cells, respectively. The BrdU assay was performed at day 2 and 4. After adding 10 µM of BrdU solution to cell culture medium, HDFs were incubated at 37 ℃ for 3 h in order to induce incorporation of BrdU into cellular DNA. Then, the subsequent procedures were performed according to the manufacturer’s instructions. The absorbance was measured using microplate reader (BioTek™ Eon™ Microplate Spectrophotometers) at 450 nm.

### Analysis of relative mRNA expression

After 3 days of the protein or protein nanoparticle treatment (20 µg/ml of control HSA NPs, 5 µg/ml of commercial bFGF, 5 µg/ml of bFGF, and 20 µg/ml of HSA-bFGF NPs, respectively), HDFs were harvested for RT-PCR analysis. Total RNA was obtained from harvested cells by NucleoSpin RNA LL kit (Macherey-Nagel, Germany), and complementary DNA (cDNA) was reversely transcribed from 1 µg RNA by RevertAid Reverse Transcriptase (thermo scientific, USA). THUNDERBIRD™ Next SYBR® qPCR Mix (TOYOBO, JAPAN) and qRT-PCR detection system (Bio-Rad CFX connect) were used for real-time RT-PCR. As a housekeeping gene, *human glyceraldehyde 3-phosphate dehydrogenase* (*GAPDH*) was used as a control for normalization. Transcript level was calculated relative to control (HSA NPs-treated) and the relative fold induction was calculated using the 2^−ΔΔCt^ algorithm. Sequences of the primers used in the real-time PCR analysis were as follows: *GAPDH* (forward 5’-ACCCACTCCTCCACCTTTGA − 3’; reverse 3’- CTGTTGCTGTAGCCAAATTCGT − 5’), *α-SMA* (forward 5’- TGGGACAAAAAGACAGCTACG − 3’; reverse 3’- GATGCCATGTTCTATCGGGTA − 5’), *TGFβ-1 (*forward 5’- AACAATTCCTGGCGATACCTC − 3’; reverse 3’- ACAACTCCGGTGACATCAAAA − 5’), *TGFβ-2* (forward 5’- TCAAGAGGGATCTAGGGTGGAA − 3’; reverse 3’- GGCATGCTCCAGCACAGAA − 5’).

### Immunocytochemical analysis

After 6 days of the protein or protein nanoparticle treatment (20 µg/ml of control HSA NPs, 5 µg/ml of commercial bFGF, 5 µg/ml of bFGF, and 20 µg/ml of HSA-bFGF NPs, respectively), immunocytochemical analysis was conducted, to investigate expression of fibrosis-related marker proteins. The HDFs were fixed with 4% paraformaldehyde diluted in PBS for 10 min at room temperature. Then, the fixed cells were permeabilized with 0.25% Triton X-100 diluted in PBS (PBST) for 10 min, and they were blocked with 3% bovine serum albumin (BSA) in 0.1% PBST for 1 h. After the fixation, permeabilization, and blocking in series, cells were incubated with primary antibodies diluted to final concentration of 1:1000 in 1% BSA in 0.1% PBST solution overnight on a rocker at 4 ℃. To investigate fibrosis, anti-α-SMA antibody (Genetex, GTX100034), anti-TGFβ-1 antibody (Bioss, BS-0086R), and anti-TGFβ-2 antibody (Bioss, BS-20412R) were used. After incubation with the primary antibodies, cells were washed with PBST, and then they were incubated with the secondary antibodies, diluted in 5% BSA in PBST at 1:1000 dilution, for 1 h at room temperature. As a secondary antibody, an anti-rabbit IgG-Alexa 488 conjugate antibody (Invitrogen, A-11,008) was utilized. Lastly, the cells were incubated with 4’,6-diamidino-2-phenylindole (DAPI) for 5 min in the dark, and then observed by confocal laser scanning microscopy (CLSM, Leica, DFC3000G).

### Cell migration assay

The HDFs were seeded at a density of 5 × 10^4^ cells/ml onto 12-well plates. When the cell confluency over 90%, the middle of a well was scratched in a straight line using a 200 µl pipette tip, followed by washing with Dulbecco’s PBS (DPBS). Then, 10 µg/ml of mitomycin C was treated with Dulbecco’s minimal essential medium (DMEM) with high glucose supplemented with 1% fetal bovine serum (FBS) and 1% penicillin-streptomycin. After incubation for 2 h, the media was removed, and cells were washed with DPBS. Then, 20 µg/ml of control HSA NPs, 5 µg/ml of commercial bFGF, 5 µg/ml of bFGF, and 20 µg/ml of HSA-bFGF NPs were treated, respectively. For wound healing assay, HDFs were observed using a microscope after 0, 12, 24, and 40 h. The cells were maintained in a humidified incubator at 37 ℃ under an atmosphere of 5% CO_2_.

### Single cell trajectory assay

For analysis of single cell migration, HDFs were seeded onto 35 mm culture dishes, and treated with 20 µg/ml of control HSA NPs, 5 µg/ml of commercial bFGF, 5 µg/ml of bFGF, and 20 µg/ml of HSA-bFGF NPs, respectively. Then, HDFs with protein or protein nanoparticles were observed for 72 h, using a phase-contrast microscope (Leica DMi8, Germany) equipped with an incubator. Phase-contrast images were taken every 10 min for time-lapse imaging. From the obtained time-lapse images, cells were manually tracked using the tracking plugin in Fiji Image J software (National Institutes of Health, USA).

### In vivo wound healing experiments

All animal experiment procedures were conducted according to the National Institute of Health (NIH) guidelines. The laboratory animal experiments and protocols were approved by the Institutional Animal Care and Use Committee (IACUC) of Ulsan National Institute of Science and Technology (UNIST). SPF SD rats (6 weeks old, male) were purchased from Orient Bio, and handled in UNIST. The rats were randomly divided into four groups for higher dose: non-treated (NT) rat as a control group (*n = 3*), HSA NPs-treated rat (*n = 5*), soluble bFGF (protein only, no nanoparticle formulation)-treated rat (*n = 5*), and HSA-bFGF NPs-treated rat (*n = 5*). The lower dose treated groups were divided into three groups: NT rat as a control group (*n = 3*), HSA NPs-treated rat (*n = 5*), and HSA-bFGF NPs-treated rat (*n = 5*). To clearly assess the wound healing and tissue regeneration process, all rats were shaved and full-thickness excisional wound (~ 1 cm in diameter) was formed on the dorsum (Fig. S2). The buffered aqueous solution (25 µl) containing soluble bFGF protein and the protein nanoparticles were directly dropped at the wounded area and dressed with commercial transparent polyurethane (PU) membranes (1624w, Tegaderm Film, 3 M) for secure protection from further injury or infection. Adhesive bandage was used to each rat for preventing potential abrading. There was no sign of unintended inflammation or infection in any of the groups. The size of wound was reduced over a period of time in all the groups, which were monitored after removing the PU membrane, and the wound size was measured by Image J software.

### Histological analysis

On the 14th day after drug administration, the wound tissue was collected and washed with PBS before being fixed in a 10% formalin solution for approximately one to two days at room temperature. Following tissue processing, the samples were embedded in paraffin and sectioned to a thickness of 5 μm. Deparaffinization and dehydration were performed, followed by hematoxylin and eosin (H&E) staining and immunofluorescence staining. For immunofluorescence staining, the anti-CD31 antibody (Abcam, ab281583), which is a marker for vascular endothelial cells, was diluted to 1:100, while the secondary antibody, anti-mouse IgG (Abcam, ab150113), was diluted to 1:200. Fluorescence imaging was conducted using a laser scanning confocal microscope (LSM780, Zeiss, Germany) and an inverted microscope (Eclipse Ti2-E, Nikon, Japn).

### In vitro angiogenic tube formation assay

The angiogenesis assay kit was purchased from Chemicon (ECM625). First, 50 µl of ECM matrix gel solution was applied to each well of a 96-well plate. The plate was then incubated at 37 ℃ for 1 h to allow the matrix solution to solidify. Next, 150 µl of endothelial cell growth medium (EGM) was added to each well of the 96-well plate. HUVECs at passage 5 were then seeded onto the surface of the polymerized ECM matrix at a density of 1 × 10^4^ cells per well followed by the experimental conditions as follows: NT, 20 µg/ml of HSA NPs, 5 µg/ml of bFGF protein, and 10 µg/ml of HSA-bFGF NPs (containing 2.5 µg/ml of bFGF protein). The experiment was repeated five times (*n = 5*). At designated time after incubation with protein or protein nanoparticles (0, 2, 4, 6, and 8 h), the wells were examined to analyze the cell sprouting under a 40X magnification using a Nikon microscope. At the extended time (9, 18, and 24 h), the observation was conducted to reveal the intercellular connections and stability at a 10X magnification with the same Nikon microscope (Fig. S5).

### CAM assay

Five-day fertilized chick embryos weighing 52–60 g were obtained from DukSan Farm (Korea). The embryos were cleaned using 70% ethyl alcohol and then pre-incubated at 37.5 ℃ with 70–80% humidity for 4 h. To prepare the embryos for experiment, 4 ml of albumin was extracted from the eggs using 5 ml needles. To prevent eggshell fragments from falling inside, the eggs were tapped gently. A window measuring 3 cm by 3 cm was carefully created using scissors, allowing for easy identification of the vascular zone on the CAM. The embryos were then treated with different agents as follows: 20 µg/ml of HSA NPs, 5 µg/ml of bFGF protein, and 20 µg/ml of HSA-bFGF NPs (containing 5 µg/ml of bFGF protein). Following the treatment, each egg was incubated at 37.5℃ with 70–80% humidity for 6 days. Photographic observations were made at day 0, 4, and 6 to capture images of the CAM and blood vessel formation.

### Statistical analysis

All the experiments were conducted in at least triplicate. Data were expressed as the mean ± standard deviation (SD). Statistical significance was determined using the t-test to compare two groups. For all experiments, statistical significance was represented by * for *p* < 0.05, ** for *p* < 0.01, and *** for *p* < 0.001.

### Electronic supplementary material

Below is the link to the electronic supplementary material.

Supplementary Material 1

## Data Availability

The data that support the findings of this study are available from the corresponding author upon reasonable request.
