# Supplementary material for: Secured delivery of basic fibroblast growth factor using human serum albumin-based protein nanoparticles for enhanced wound healing and regeneration
Source: J Nanobiotechnology. 2023 Sep 2;21:310. doi: 10.1186/s12951-023-02053-4 (PMC10474766; doi:10.1186/s12951-023-02053-4)
Supplement: Supplementary file 1 — Supplementary Material 1 [file 12951_2023_2053_MOESM1_ESM.docx]

***Journal of Nanobiotechnology***

***Supplementary Material***

**Secured delivery of basic fibroblast growth factor using human serum albumin-based protein nanoparticles for enhanced wound healing and regeneration**

Boram Son^1,#^, Minju Kim^3,#^, Hyosub Won^1,#^, Ara Jung^4,5^, Jihyun Kim^1^, Yonghoe Koo^3^, Na Kyeong Lee^6^, Seung-Ho Baek^7^, Uiyoung Han^8^, Chun Gwon Park^9^, Heungsoo Shin^1^, Bomi Gweon^4,^*, Jinmyoung Joo^3,^* and Hee Ho Park^1,2,^*

^1^Department of Bioengineering, Hanyang University, Seoul, Republic of Korea.

^2^Research Institute for Convergence of Basic Science, Hanyang University, Seoul, Republic of Korea.

^3^Department of Biomedical Engineering, Ulsan National Institute of Science and Technology (UNIST), Ulsan, Republic of Korea.

^4^Department of Mechanical Engineering, Sejong University, Seoul, Republic of Korea.

^5^Department of Biomedicine & Health Science, College of Medicine, The Catholic University of Korea, Seoul, Korea.

^6^Department of Intelligent Precision Healthcare Convergence, Sungkyunkwan University (SKKU), Suwon, Republic of Korea.

^7^Center for Bio-based Chemistry, Korea Research Institute of Chemical Technology (KRICT), Ulsan, Korea.

^8^Department of Ophthalmology, Stanford University School of Medicine, Stanford, CA, USA.

^9^Department of Biomedical Engineering, SKKU Institute for Convergence, Sungkyunkwan University (SKKU), Suwon, Republic of Korea.

^#^These authors contributed equally.

*To whom correspondence should be addressed.

Professor Bomi Gweon, Ph.D., Tel.: +82 2 3408 3114, Email: [bgweon@sejong.ac.kr](mailto:bgweon@sejong.ac.kr)

Professor Jinmyoung Joo, Ph.D., Tel.: +82 52 217 3251, Email: [jjoo@unist.ac.kr](mailto:jjoo@unist.ac.kr)

Professor Hee Ho Park, Ph.D., Tel.: +82 2 2220 0497, Email: [parkhh@hanyang.ac.kr](mailto:parkhh@hanyang.ac.kr)


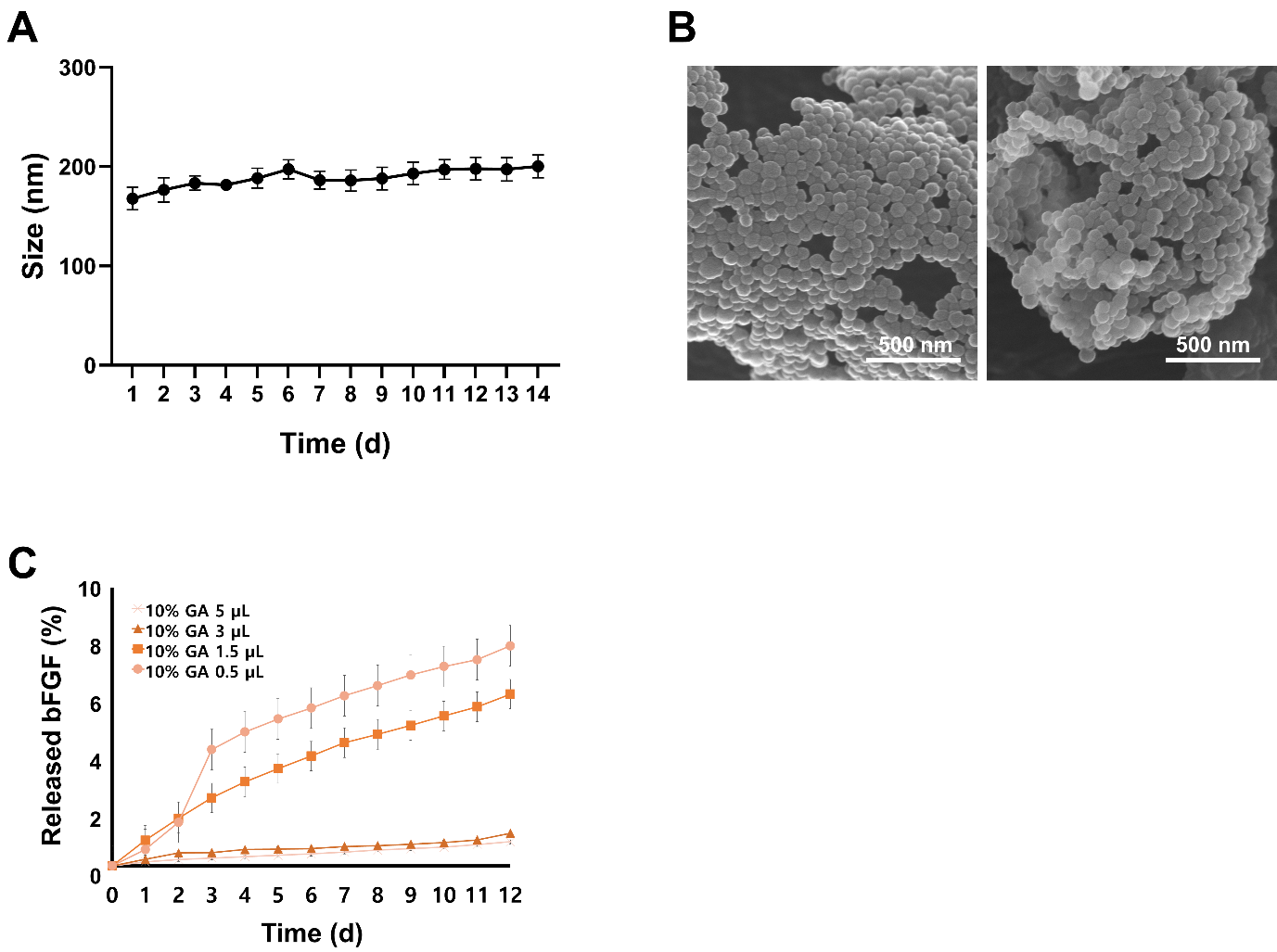


**Fig. S1. Long term characteristics of human serum albumin (HSA)-basic fibroblast growth factor (bFGF) protein nanoparticles (NPs).** (A) Size of HSA-bFGF NPs depending upon incubation time (up to 14 days). (B) Scanning electron microscope (SEM) images of HSA-bFGF NPs after 14-day incubation. Scale bars, 500 nm. (C) *In vitro* release profile (%) of bFGF from HSA-bFGF NPs depending upon the amount of added crosslinker, glutaraldehyde (GA).


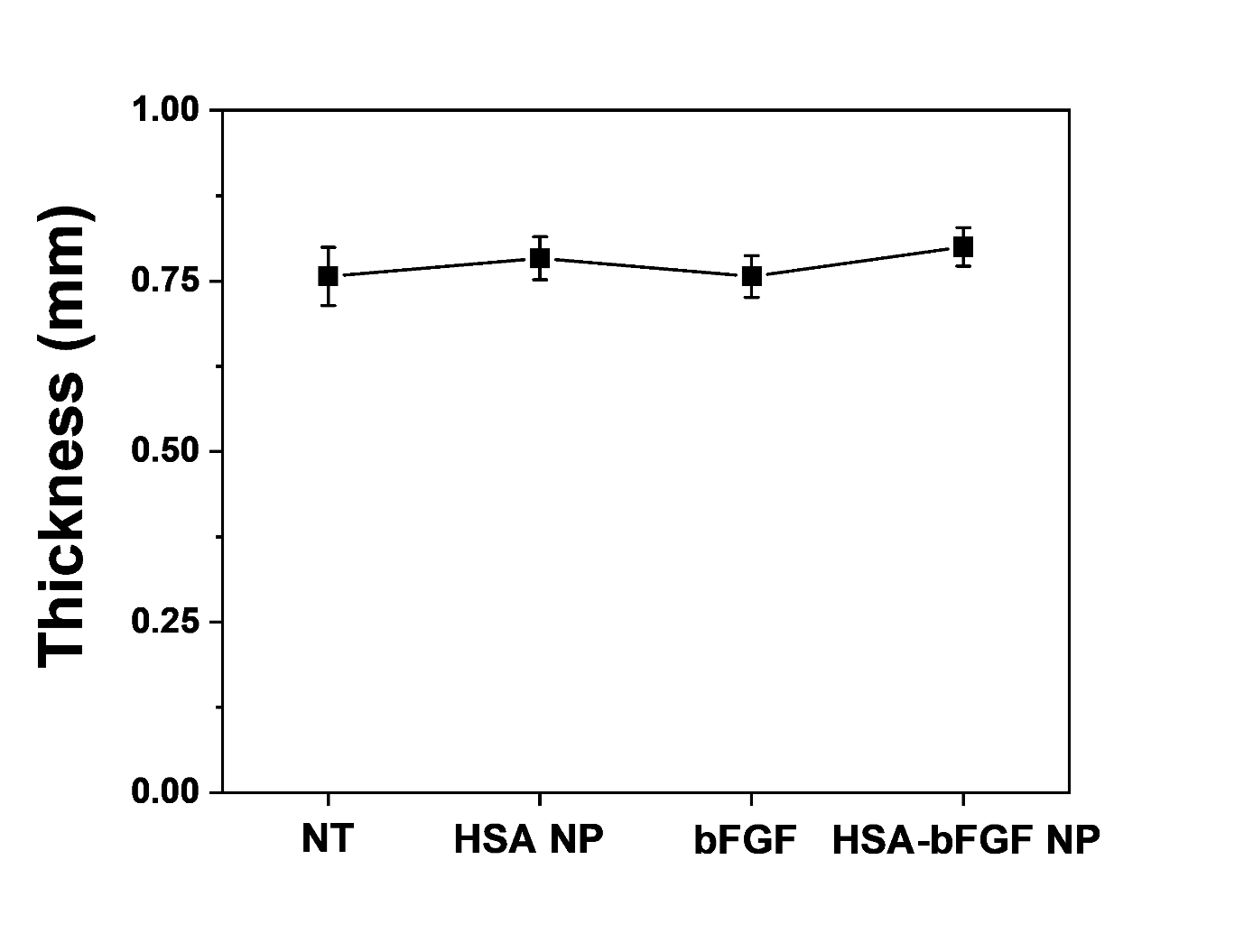


**Fig. S2. Thickness of excised skin tissue.** Comparable skin tissue thickness obtained from the wound model of rat shows consistent and uniform formation of excisional wound in the present study.

**
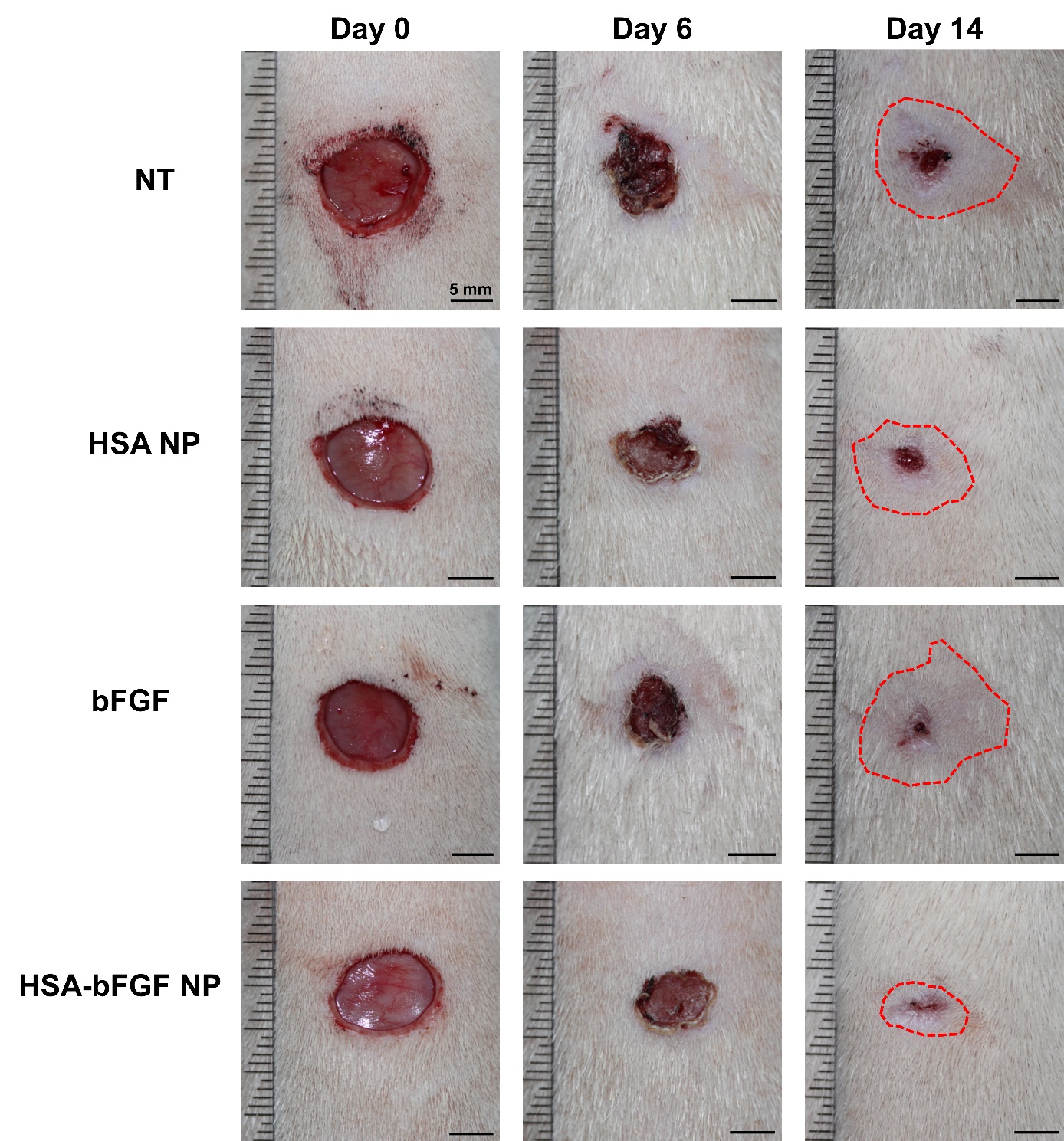
**

**Fig. S3. Magnified photographs of the rat skin after wound healing process.** Representative magnified photographs of the rat skin wound healing process treated with HSA NPs, soluble bFGF and HSA-bFGF NPs, compared to non-treated group (NT) at day 0, 6 and 14. The red dotted lines indicate the areas where hair has visibly regrown. Among the treatment groups, the group treated with HSA-bFGF NPs demonstrated the most pronounced hair growth. Scale bars, 5mm.

**
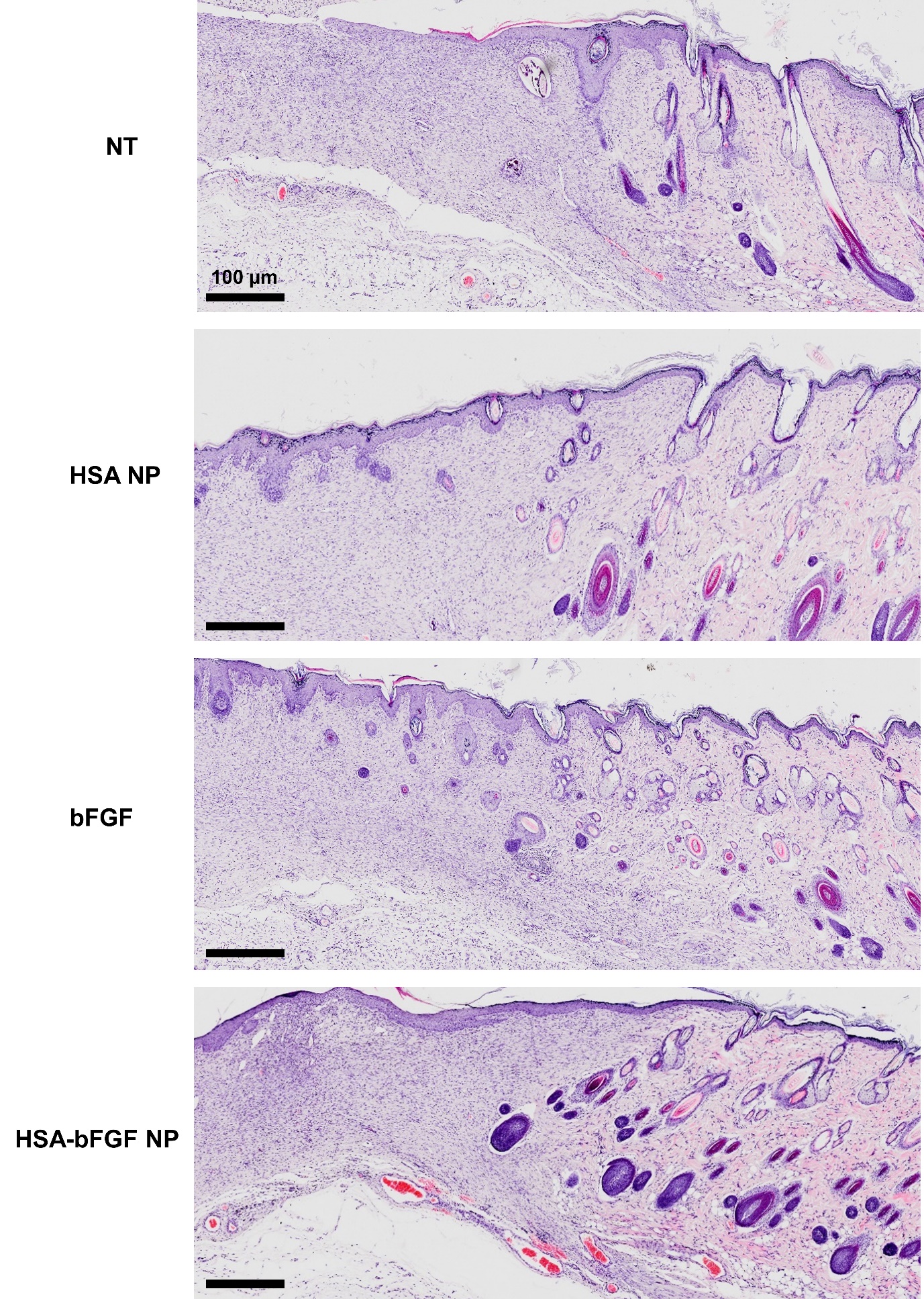
**

**Fig. S4. Magnified histological tissue analysis.** Representative magnified hematoxylin & eosin (H&E) staining images of each skin treated with HSA NPs, soluble bFGF and HSA-bFGF NPs, compared to NT. Enhanced angiogenesis was observed in the tissue treated with HSA-bFGF NPs at the wound site. Scale bars, 100 μm.

**
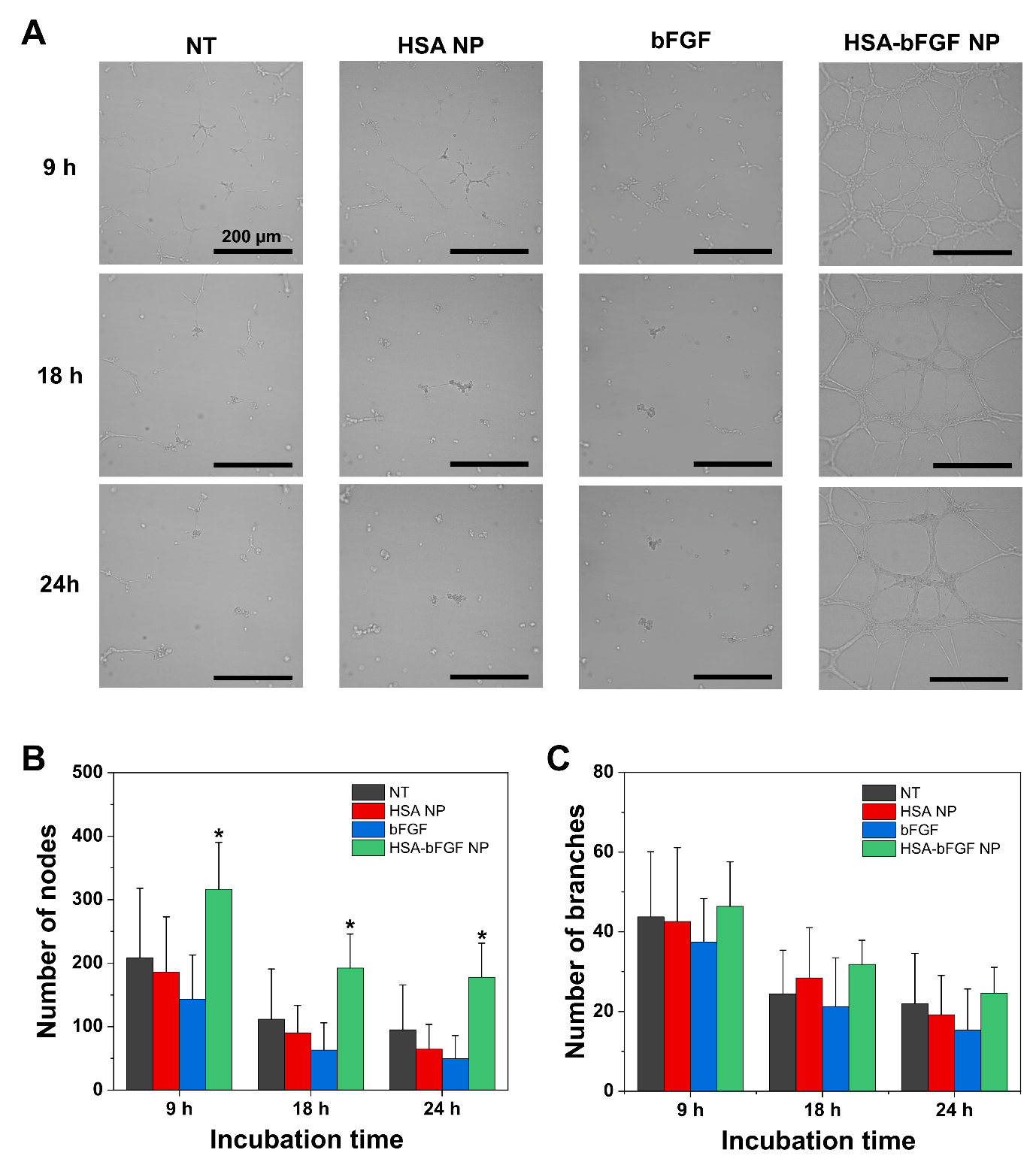
**

**Fig. S5. Intercellular connections and angiogenesis of human umbilical human umbilical vein endothelial cells (HUVECs) *in vitro*.** (A) Representative images showing angiogenic feature of HUVECs upon treatment with HSA NPs, soluble bFGF and HSA-bFGF NPs, compared to NT. The HSA-bFGF NPs exhibited the superior property to form and maintain blood vessels compared to other groups. Scale bars, 200 μm. (B, C) Quantitative analysis of angiogenesis parameters such as number of nodes (B), and branches (C). Cells treated with HSA-bFGF NPs demonstrated significantly higher node counts, indicating their beneficial effect on angiogenesis correspondingly. * *p* < 0.05.
